# Clinical Presentation and Outcome of Acute Respiratory Illnesses in South African Children During the COVID-19 Pandemic

**DOI:** 10.1097/INF.0000000000003951

**Published:** 2023-07-13

**Authors:** Andrew Redfern, Marieke M. van der Zalm, Juanita Lishman, Pierre Goussard, Liezl Smit, Ron Dagan, Mikhail Barday, Minette Mare, Mathilda Claassen, Gert Van Zyl, Helena Rabie, Lilly M. Verhagen

**Affiliations:** From the * Department of Paediatrics and Child Health, Tygerberg Hospital and Stellenbosch University, Cape Town, South Africa; † Desmond Tutu Tuberculosis Centre, Department of Paediatrics and Child Health, Faculty of Medicine and Health Sciences, Stellenbosch University, Cape Town, South Africa; ‡ The Faculty of Health Sciences, Ben-Gurion University of the Negev, Beer-Sheva, Israel; § Division of Medical Virology, Stellenbosch University and National Health Laboratory Services, Cape Town, South Africa; ¶ Laboratory of Medical Immunology, Radboud Institute for Molecular Life Sciences, Radboud University Medical Center, Nijmegen, The Netherlands; ‖ Department of Paediatric Infectious Diseases and Immunology, Amalia Children’s Hospital, Radboud Center for Infectious Diseases, Radboud University Medical Center, Nijmegen, The Netherlands.

**Keywords:** COVID-19, SARS-CoV-2, pneumonia, respiratory tract infection, pediatrics, children

## Abstract

**Background:**

Data from low- and middle-income countries (LMICs) show higher morbidity and mortality in children with acute respiratory illness (ARI) from severe acute respiratory syndrome coronavirus-2 (SARS-CoV-2). However, whether SARS-CoV-2 infection is distinct from other causes of ARI in this regard is unclear. We describe clinical characteristics and outcomes of South African children with SARS-CoV-2 and non-SARS-CoV-2 ARIs.

**Methods:**

We performed a cross-sectional study including 0–13 years old children admitted to Tygerberg Hospital between May and December 2020 with an ARI. Routine clinical data were collected by the attending clinicians. All children underwent SARS-CoV-2 polymerase chain reaction testing. For severity of disease, the need for respiratory support and duration of support was considered. Multivariable logistic regression models were built to determine the factors associated with SARS-CoV-2 infection and severity.

**Results:**

Data for 176 children were available, 38 (22%) children were SARS-CoV-2 polymerase chain reaction positive and 138 (78%) were negative. SARS-CoV-2 positive children were more likely to be female (OR: 2.68, 95% CI: 1.18–6.07), had lower weight-for-age Z score (OR: 0.76, 95% CI: 0.63–0.93), presented more frequently with fever (OR: 3.56, 95% CI: 1.54–8.24) and less often with cough (OR: 0.27, 95% CI: 0.11–0.66). SARS-CoV-2 infection was associated with significantly longer duration of oxygen treatment (median 8 vs. 3 days; OR: 1.1, 95% CI: 1.01–1.20). Overall, 66% of children had viral coinfection, with no significant difference between the groups. In total, 18% of SARS-CoV-2 positive children were readmitted within 3 months for a respiratory reason, compared with 15% SARS-CoV-2 negative children (*P* = 0.64).

**Conclusions:**

Our data show that ARIs from SARS-CoV-2 cannot be easily differentiated, but were associated with a higher morbidity compared with ARIs from other causes. Overall outcomes were good. The long-term implications of severe SARS-CoV-2 pneumonia in young children in low- and middle-income countries require further study.

In low- and middle-income countries (LMICs) such as South Africa, the burden of acute respiratory illnesses (ARIs) is high, and is associated with significant morbidity and mortality, especially in children under 5 years of age.^1,2^ The social-economic and healthcare context, as well as epidemiological and clinical risk factors such as HIV, tuberculosis (TB) and undernutrition, may influence the clinical presentation and outcomes of ARIs in these settings. Initial data suggest that severe acute respiratory syndrome coronavirus-2 (SARS-CoV-2) in children in LMICs generally has higher morbidity and mortality compared with the high-income countries (HICs).^3–8^ However, it remains unclear whether SARS-CoV-2 is similar in presentation and outcome to ARIs from other causes in children in LMICs.

Data from across the world, including surveillance data from South Africa, revealed marked changes in epidemiological profile of seasonal respiratory viruses over the course of the coronavirus disease 2019 (COVID-19) pandemic, with lower detection rates of influenza and respiratory syncytial virus (RSV) particularly, and a relative increase in human rhinovirus (HRV).^9–11^

Rates of viral coinfection with SARS-CoV-2 in children vary greatly in the literature, from rates as low as 1.8% up to 26%.^3,12^ The wide variation may be partly explained by patient selection, lack of routine testing and epidemiological changes during the course of the pandemic. Children may have higher rates of coinfection compared with adults, and this may be associated with increased severity of illness.^13^

In this study, we compared the clinical presentation and outcomes of children with polymerase chain reaction (PCR)-confirmed SARS-CoV-2 infection with those who had a negative PCR among children admitted with an ARI. In addition, we investigated viral coinfection with other respiratory viruses.

## METHODS

### Study Design

We performed a cross-sectional study including 0- to 13-year-old children, who were admitted to Tygerberg Hospital between May 5, 2020 (week 19) and December 5, 2020 (week 48) with an ARI. This period falls within the beginning of autumn until the beginning of summer. South Africa reported its first case of SARS-CoV-2 on March 5, 2020, with the first wave officially starting at the beginning of May 2020 in the Western Cape (week 19).^14^

### Setting

Tygerberg Hospital is a large public tertiary hospital in the Western Cape province of South Africa providing specialist and subspecialist pediatric services. General pediatric services in South Africa provide care for children up to the age of 13 years, which is the rationale for the age cutoff for our study population. The majority of patients accessing the hospital come from poor socio-economic circumstances. Due to limited critical care beds, respiratory support in the form of nasal continuous positive airway pressure using Fisher and Paykel variable flow system, or high-flow nasal cannula oxygen therapy using the Airvo Optiflow system are mostly provided in the pediatric emergency unit and general pediatric wards.

### Study Participants and Definitions

Children admitted with symptoms of an ARI and who had a SARS-CoV-2 PCR test were included. ARIs were classified into one of the 3 diagnostic categories according to the main presenting feature, namely upper respiratory tract infection (URTI), lower respiratory tract infection (LRTI) or asthma/wheeze. URTI was defined as having at least 1 respiratory symptom, without any signs of tachypnea or respiratory distress. LRTI was defined as any child with respiratory symptom(s) and evidence of tachypnea or respiratory distress, including acute viral bronchiolitis. Asthma (>5 years) or wheeze (≤5 years) were classified if the predominant clinical presentation was wheeze/reversible bronchospasm. We excluded babies admitted to the neonatal service, children whose SARS-CoV-2 PCR was positive but who did not have any respiratory symptoms, and children in whom a SARS-CoV-2 PCR result was not available. Children with multisystem inflammatory syndrome in children were only included if their presentation was as an ARI.

### Data Collection

A standard data collection form was used to capture routine clinical and laboratory information at the time of presentation to pediatric emergency unit or admission to a ward. All cases were reviewed 3–6 months after the initial admission to assess mid-term outcome and record any readmissions within 3 months of initial presentation. Missing information was subsequently retrospectively captured using patient clinical records and the National Health Laboratory system. Unfortunately, in cases where the data collection form was not fully completed at the time of admission, certain data were missing despite retrospective review of the medical records, and consequently not all variables were captured for all cases. De-identified data were captured onto a secure RedCap database. A waiver of individual consent was obtained from Stellenbosch University Human Research Ethics committee (HREC N20/04/013_COVID). Data of some children were previously published.^6,7^

De-identified digital chest radiographs (CXR) were reported by 2 external expert reviewers, who were blinded to any clinical information apart from age. Radiological features were described in accordance with World Health Organization classification of pneumonia as either alveolar infiltrate, other infiltrate (which included interstitial infiltrates) or no pneumonia.^15^ Weight-for-age Z-scores (WAZ) were calculated using a World Health Organization-based app developed by the Canadian Pediatric Endocrine group.^16^

Throughout the study period, all children admitted with ARI symptoms underwent real-time reverse-transcription polymerase chain reaction testing for SARS-CoV-2 via a single nasopharyngeal swab as part of routine care. Testing for other respiratory viruses was not performed routinely, but at the discretion of the clinical team or retrospectively as part of the study if samples were still available. Tests were performed in a registered virology laboratory using Allplex 2019-nCoV assay (Seegene Inc) and manufacturer’s cycle threshold cutoff values were used. Multiplex PCR testing for respiratory viral panel was performed using either the Anyplex II RV16 detection assay or the Allplex RV Essential Assay (both from Seegene Inc). Epidemiologic data from the National Institute of Communicable disease shows that the Ancestral type variant (May 3, 2020 to August 16, 2020) and Beta variant (November 8, 2020 to February 7, 2021) were present during the study period.^17^

### Data Analysis

Data analysis was done using IBM SPSS Statistics v27. The χ^2^ test was used to compare categorical variables, and independent samples *t* test or Mann-Whitney *U* test were used to compare continuous variables. Multivariable analysis was performed using logistic regression models to study the association of demographic/clinical variables with SARS-CoV-2 infection and severity. Variables included in multivariable analysis model assessing SARS-CoV-2 positivity were age, sex, WAZ, any comorbidity, COVID contact, HIV exposure or disease, TB exposure or disease and reported symptoms and signs as listed in Table 2. Variables included in the model assessing severity and outcome were age, gender, need for oxygen or further respiratory support, need for pediatric intensive care unit (PICU) admission, length of stay and number of days on oxygen. Level of significance was set at 0.05. Laboratory markers were excluded from the multivariable models because of a high percentage of missing data.

## RESULTS

A total of 176 children were included in our study, of which 38 (21.6%) were SARS-CoV-2 PCR positive and 138 (78.4%) were SARS-CoV-2 PCR negative.

### Patient Characteristics

Patient characteristics are shown in Table 1. Nearly half of all patients were <1 year of age. There were proportionately more infants in the SARS-CoV-2 positive group (65% vs. 41%, *P* = 0.01). Compared with SARS-CoV-2 negative children, children with SARS-CoV-2 infection were younger (median 6.6 months vs. 17.0 months, *P* = 0.03) and tended to be female (55% vs. 38%, *P* = 0.06). Nearly one third of all children had comorbidities (30%), but there was no significant difference between the SARS-CoV-2 positive and negative groups (26% vs. 31%, *P* = 0.56). The most frequent comorbidities were premature birth (19%), HIV exposure (29/143, 20%), HIV positive (9/149, 6%), 10 of 167 (6%) with current or previous TB disease, 7 of 176 (4%) known with asthma, 6 of 176 (3%) with cancer and 5 of 176 (3%) with a cardiac condition.

**TABLE 1.** Demographic Characteristics of Patients

	n	All (n = 176)	SARS-CoV-2 Positive (n = 38)	SARS-CoV-2 Negative (n = 138)	*P* Value
Age (median, IQR) in months	176	14.1 (3.4–40.6)	6.6 (2.1–23.7)	17.0 (4.6–42.0)	0.03
Age categories					0.08
0–2 mo		42 (24%)	14 (37%)	28 (20%)	
3–11 mo		39 (22%)	11 (29%)	28 (20%)	
12–59 mo		71 (40%)	10 (26%)	61 (44%)	
>60 mo		24 (14%)	3 (8%)	21 (13%)	
Infant age group	176				0.01
<1 y		81 (45%)	25 (63%)	56 (41%)	
>1 y		95 (55%)	15 (37%)	82 (59%)	
Sex	176				0.06
Male		102 (58%)	17 (45%)	85 (62%)	
Female		74 (42%)	21 (55%)	53 (38%)	
Premature birth	160	31 (18%)	7/34 (21%)	24/126 (19%)	0.92
WAZ (median, IQR)	176	−0.62 (−1.7 to 0.34)	−0.83 (−2.5 to −0.11)	−0.54 (−1.6 to −0.44)	0.03
Any comorbidity*	176	53 (30%)	10 (26%)	43 (31%)	0.56
HIV exposed (%)	143	29 (20%)	8/30 (27%)	21/111 (19%)	0.72
HIV status	149				0.64
HIV uninfected		140 (94%)	30/31 (97%)	110/118 (93%)	
HIV infected		9 (6%)	1/31 (3)%	8/118 (7%)	
TB exposure	167				0.53
Not TB exposed		152 (91%)	34/35 (97%)	120/132(90%)	
TB exposed		15 (9%)	3/35 (8%)	12/132 (9%)	
TB disease	173				0.74
Never TB disease		163 (94%)	35/37 (95%)	128/136 (94%)	
Current TB disease		7 (4%)	2 (5%)	5 (4%)	
Previous TB disease		3 (2%)	0	3 (2%)	
Known/suspected COVID contact^†^	176	17 (10%)	6 (16%)	11 (8%)	0.15

* This includes children with current TB disease (7), HIV infected (9), asthma (7), cardiac (5), oncological (6), cerebral palsy (4). Selected other conditions included one child with diabetes and two children with sickle cell disease.

† Known COVID contact was defined as having been in contact with a confirmed COVID-19 case. A suspected COVID contact was defined as having been in contact with a person meeting the case definition for COVID-19, but whose SARS-CoV-2 status was unknown.

### Clinical Presentation

Comparison of the clinical features, special investigations, treatment and outcomes in SARS-CoV-2 positive and negative children is shown in Table 2. In univariable analysis, measured or reported fever was more common in children with SARS-CoV-2 infection compared with those without (68% vs. 40%; *P* = 0.01). Children with SARS-CoV-2 were less likely to present with cough (63% vs. 85%; *P* = 0.01), tight chest (48% vs. 70%; *P* = 0.02) or wheeze (33% vs. 51%; *P* = 0.03). There were no other symptoms or signs, which were significantly different between the groups.

**TABLE 2.** Comparison of the Clinical Presentation, Management and Outcome of SARS-CoV-2 Positive and Negative Children

Symptoms	n	All (n = 176)	SARS-CoV-2 Positive (n = 38)	SARS-CoV-2 Negative (n = 138)	*P* Value
Fever*	176	81 (46%)	25 (68%)	56 (40%)	0.01
Cough^†^	176	142 (80%)	25 (63%)	117 (85%)	0.01
Abdominal symptoms^‡^	176	48 (27%)	13 (33%)	35 (25%)	0.28
Runny nose/rhinorrhea	176	88(50%)	20 (53%)	68 (49%)	0.72
Tight chest	176	116 (65%)	19 (48%)	97 (70%)	0.02
Headache	176	9 (5%)	3 (8%)	6 (4%)	0.33
Examination features					
Hypoxia (<92%)	155	60 (38%)	14/32 (41%)	46/123 (37%)	0.58
Tachypnea^§^	176	105 (59%)	21 (53%)	84 (61%)	0.34
Tachycardia ^¶^	176	80 (46%)	16 (45%)	64 (46%)	0.64
Increased work of breathing	176	129 (72%)	29 (73%)	100 (72%)	0.64
Wheeze	176	83 (47%)	12 (33%)	71 (51%)	0.03
Crepitations	176	95 (53%)	17 (43%)	78 (57%)	0.20
Stridor	176	8 (5%)	2 (5%)	6 (4%)	0.57
Investigations					
CRP (median, IQR)	141	11 (2–35)	14 (2–25)	10 (2–37)	0.93
White cell count (median × 10^9^/L, IQR)	140	12 (8.1–16.6)	12.7 (10.4–19.0)	11.9 (7.7–16.6)	0.33
Lymphocyte count (median × 10^9^/L)	120	3.1 (1.8–4.8)	3.8 (1.7–6.3)	2.8 (1.8–4.5)	0.32
Platelet count (mean × 10^9^/L)	136	411	422	401	0.56
Blood culture positive	88	10 (12%)	3/24 (13%)	7/64 (11%)	0.20
Chest radiography^‖^					
Alveolar infiltrate	162	57 (35.2%)	18 (48.6%)	39 (31.2%)	0.06
No pneumonia	162	103 (63.6%)	19 (51.4%)	84 (67.2%)	
Other infiltrates	162	1 (0%)	0 (0%)	1 (0.7%)	–
Diagnosis					
URTI	176	32 (18%)	7 (20%)	25 (18%)	0.17
LRTI	176	133 (75%)	31 (80%)	102 (74%)	0.43
Asthma/viral wheeze	176	16 (9%)	1 (3%)	15 (11%)	0.20
Management					
Nasal prong oxygen (2L/min or less)	176	121 (68%)	27 (68%)	94 (68%)	0.73
High-flow (HF)	176	48 (27%)	15 (38%)	33 (24%)	0.06
CPAP	176	14 (8%)	6 (15%)	8 (6%)	0.06
Invasive ventilation (IPPV)	176	11 (6%)	3 (8%)	8 (6%)	0.64
Respiratory support (HF/CPAP/IPPV)	176	51 (28%)	15 (38%)	36 (26%)	0.11
Any oxygen supplementation	176	132 (74%)	29 (76%)	103 (75%)	0.83
Oxygen duration (median, IQR)	132	2 (1.0-6.0)	6 (1.0–9.2)	2 (1.0–4.5)	0.01
Dexamethasone	176	8 (4.5%)	4 (10.5%)	4 (2.9%)	0.07
Prednisone	176	65 (36.9%)	17 (44.7%)	48 (34.8%)	0.26
Metered dose inhaler	176	64 (36.4%)	10 (26.3%)	54 (39.1%)	0.15
Nebulization	176	45 (25.6%)	6 (15.8%)	39 (28.3%)	0.12
Intravenous antibiotics	176	98 (55.7%)	25 (65.8%)	73 (52.9%)	0.16
Oral antibiotics	176	128 (72.7%)	25 (65.8%)	103 (74.6%)	0.28
Outcome					
PICU admission	176	16 (9%)	7 (18%)	9 (7%)	0.03
Length of stay (median days, range)	167	3.0 (0–40)	7.0 (2.0–15.0)	3.0 (2.0–8.0)	0.01
Readmission for respiratory reason	176	27 (15%)	7 (18%)	20 (15%)	0.64

* History of fever or measured fever.

† History of cough or observed cough.

‡ Includes diarrhea, vomiting and abdominal pain.

§ Tachypnea was defined as follows: 0–2 months respiratory rate (RR) > 60, 3–11 months RR > 50, 12–59 months RR > 40 and >60 months RR > 25.

¶ Tachycardia was defined as follows: 0–2 months heart rate (HR) > 170, 3–11 months HR > 160, 12–59 months HR > 150 and >60 months HR > 120.

‖ One child in the SARS-CoV-2 negative group had an radiograph which was uninterpretable due to technical issues.

CPAP indicates nasal continuous positive airway pressure.

The majority of children were diagnosed with LRTI (133/176), with a minority presenting with URTI (32/176), asthma (13/176) or viral wheeze (3/178). There was no significant difference in type of ARI between SARS-CoV-2 positive and negative children.

Using a stepwise backward logistic regression model looking at demographic and clinical features (Table 3), children with SARS-CoV-2 had statistically significantly lower weight-for-age Z-scores [odds ratio (OR): 0.76, 95% confidence interval (CI): 0.63–0.93], were more likely to be female (OR: 2.68, 95% CI: 1.19–6.07) and were more likely to have fever (OR: 3.56, 95% CI: 1.54–8.24) but less likely to have cough (OR: 0.27, 95% CI: 0.11–0.66).

**TABLE 3.** Significant Variables Associated With SARS-CoV-2 After Multivariable Analysis Using Stepwise Backward Logistic Regression Model*

Variable	OR	95% CI Lower	95% CI Upper
WAZ	0.77	0.63	0.93
Gender	2.68	1.18	6.07
Fever	3.56	1.54	8.23
Cough	0.27	0.11	0.66

* Variables included in multivariable analysis were age, sex, WAZ, any comorbidity, COVID contact, HIV exposure or disease, tuberculosis exposure or disease, as well as reported symptoms and signs as listed in Table 2.

### Severity of Disease and Treatment

A large percentage of children required oxygen supplementation (132/176, 74%) or further respiratory support in the form of high-flow, continuous positive airway pressure or invasive positive pressure ventilatin (IPPV) (51/136, 28%), in keeping with their need for admission at a referral hospital. SARS-CoV-2 positive children were not more likely to need oxygen (76% vs. 75%, *P* = 0.83) or respiratory support (38% vs. 26%, *P* = 0.11), but were more likely to require intensive care admission (18% vs. 7%, *P* = 0.03), required oxygen supplementation for longer (median 6 vs. 2 days, *P* = 0.01) and had significantly longer hospital stay (median 7 vs. 3 days, *P* = 0.01). Multivariable logistic regression analysis was performed, which included age, gender, need for oxygen or further respiratory support, PICU admission, length of stay and number of days on oxygen. This showed number of days on oxygen (OR: 1.1, 95% CI: 1.01–1.20) as the only factor independently associated with SARS-CoV-2 infection. In terms of treatment received, steroid therapy, antibiotics and bronchodilator therapy were similar between the groups.

### Special Investigations

**FIGURE 1.**
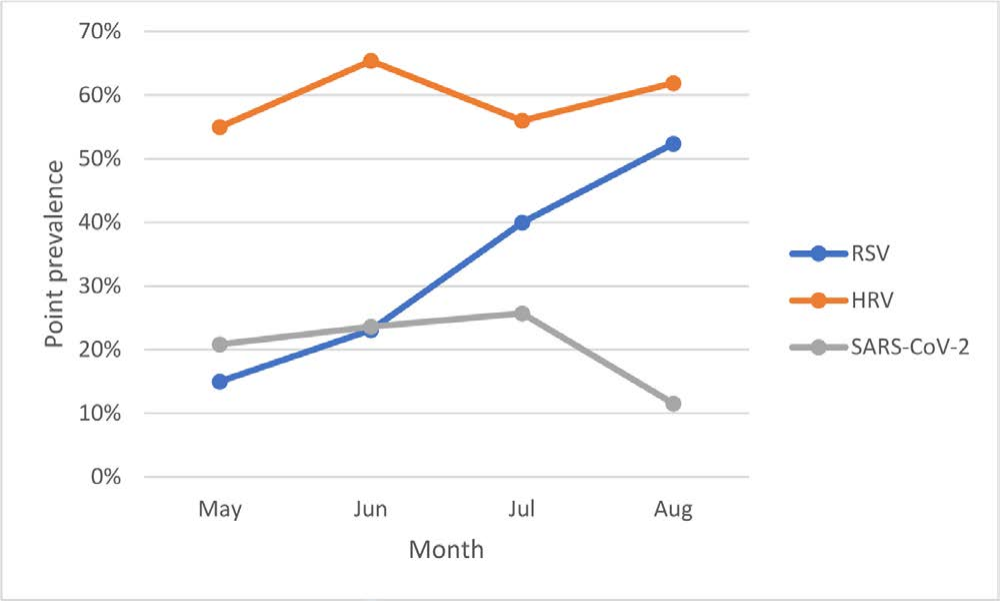
The point prevalence of Respiratory Syncytial virus (RSV), Human Rhinovirus (HRV) and SARS-CoV-2 during the winter months is shown. The number of SARS-CoV-2 and respiratory virus panel tests performed per month, in chronological order, were as follows: SARS-CoV-2 PCR/(Respiratory viruses) 48/(20), 55/(26), 35/(25) and 26/(21).

There was no difference in laboratory features between the SARS-CoV-2 positive and negative groups. Blood cultures were performed in 88 patients, with 10 being positive. Organisms grown on blood culture included coagulase-negative staphylococci (6), methicillin-sensitive *Staphylococcus aureus* (1), methicillin-resistant *S. aureus* (1), multidrug-resistant *Enterobacter cloacae* (1) and *Streptococcus mitis/oralis* (1). Alveolar infiltrates on CXR tended toward being more frequent in the SARS-CoV-2 positive group (48.6% vs. 31.2%, *P* = 0.06). Respiratory viral panels were available for 108 of 176 (61.2%) patients, and the results are shown in Table 4. Overall, another respiratory virus was detected in 71 of 108 (66%) of patients, but there was no significant difference between the SARS-CoV-2 positive and negative groups (15/27 vs. 56/81, *P* = 0.20). The most common viruses detected were HRV (56/108, 51.9%), RSV A/B (30/108, 27.8%) and human adenovirus (10/108, 9.3%). HRV point prevalence was high and consistent throughout the winter months (May to August), whereas RSV showed a consistent gradual increase over the same time period, shown in Figure 1. There was only 1 case each of influenza, parainfluenza and human metapneumovirus.

**TABLE 4.** Results of Respiratory Viral Panel Tests

	All (n = 108) N, %	SARS-CoV-2 Positive (n = 27) N, %	SARS-CoV-2 Negative (n = 81) N, %	*P* Value
Human rhinovirus	56 (51.9%)	9 (33.3%)	47 (58.0%)	0.03
RSV A/B	30 (27.8%)	6 (22.2%)	24 (29.6%)	0.46
Adenovirus	10 (9.3%)	5 (18.5%)	5 (6.2%)	0.06
Human enterovirus	2 (1.9%)	1 (3.7%)	1 (1.2%)	0.41
Human Bocavirus	3 (2.8%)	1 (3.7%)	2 (2.5%)	0.74
HCoV-229E	-	-	-	-
HCoV-NL-63	-	-	-	-
Influenza A/B	1 (0.9%)	1 (3.7%)	-	-
Parainfluenza	1 (0.9%)	-	1 (1.2%)	-
Human metapneumovirus	1 (0.9%)	-	1 (1.2%)	-
Other respiratory virus detected	71 (65.7%)	15 (55.6%)	56 (69.1%)	0.20
Single respiratory virus	44 (40.7%)	7 (25.9%)	37 (45.7%)	0.80
Multiple respiratory viruses	27 (25.0%)	8 (29.6%)	19 (23.5%)	0.52

### Clinical Outcomes

There were no deaths, apart from a child with acute myeloid leukemia who died of complications related to his underlying condition 3 months after the initial presentation. A total of 27 children were readmitted within 3 months for a respiratory reason, but there was no significant difference between SARS-CoV-2 positive and negative groups (18% vs. 15%, *P* = 0.64). Of the readmitted children who were initially SARS-CoV-2 negative, 17 of 20 had a repeat SARS-CoV-2 PCR upon readmission, of which none were positive. Of the 7 children readmitted who were initially SARS-CoV-2 positive, 2 tested SARS-CoV-2 PCR positive again between 1 and 3 months later.

## DISCUSSION

Our data in hospitalized South African children show that SARS-CoV-2-associated ARI was associated with younger age, female sex and lower WAZ score, and had higher morbidity compared with ARI in SARS-CoV-2 negative children. Although we found that SARS-CoV-2 positive children presented more frequently with fever, but less frequently with cough and wheeze, they cannot be easily distinguished based on the clinical presentation. This has implications for hospital testing and isolation strategies during future waves.

Nearly one third of all children admitted with ARI had comorbid conditions, which was similar between the groups. In total, 10 children had current TB disease or history of TB in this study, including 2 children with SARS-CoV-2 infection. Adult data show that a history of TB is a risk factor for more severe disease, but our numbers were too small to analyze this further.^18,19^ Although currently data in children is lacking, the potential impact of the COVID-19 pandemic on disruption of TB national control programs, as well as the potential for more severe TB or COVID-19, means that this is an important area for further research. No other studies, as far as we are aware, have reported lower WAZ score in children with SARS-CoV-2. The reasons for this are not clear. The lower WAZ score may reflect poorer socio-economic circumstances, and SARS-CoV-2 may spread more in overcrowded areas where there is potentially less ability to isolate and adhere to control measures, but this would probably apply to other respiratory pathogens too.

We found no difference in laboratory parameters, in contrast to Jimenez-Garcia et al and other studies of COVID-19 in children.^3,6,20^ The reason for this may be the young median age of our cohort or the high prevalence of coinfection. We found alveolar infiltrates in 48% of SARS-CoV-2 positive children, compared with 31% who were negative, and no interstitial infiltrates in the positive group. Ground glass infiltrates and consolidation are the commonest radiographical findings reported in children with SARS-CoV-2. Toba et al^3^ reported CXR findings of interstitial infiltrates in 18% of SARS-CoV-2 children and consolidation in 15%. The reason for lack of finding interstitial infiltrates in our study is not clear.

Severity of the disease and outcomes were worse in the SARS-CoV-2 positive children. They tended to require more respiratory support and had increased PICU admission. Most notably, children with SARS-CoV-2 were significantly longer on oxygen compared with SARS-CoV-2 negative children, as well as having an increased length of hospital stay. This is consistent with our previous findings, which showed that SARS-CoV-2 infection in younger children was associated with hospital admission and need for respiratory support, but this also highlights the difference between SARS-CoV-2 positive and negative children.^7^ Although SARS-CoV-2 infections have been generally reported as mild in children, our data show that there is substantial morbidity in children. Jimenez-Garcia et al^21^ also found longer oxygen duration among SARS-CoV-2 positive children, but no increased length of stay. This high morbidity may be partly explained by the lower median age of the SARS-CoV-2 positive group. In contrast to most studies of pediatric COVID-19, nearly half of our study population were infants, and infants were more likely to be SARS-CoV-2 positive.^3,8,20,22^ There is increasing evidence that young age is a risk factor for severe disease from SARS-CoV-2. Bhuiyan et al showed that half of “young COVID-19” (ie, <years) cases occurred in infants, with 7% of these cases requiring intensive care unit admission.^22^ Graff et al reported that infants <3 months of age with COVID-19 were more likely to require admission.^23^ A large study in the 6 sub-Saharan African countries of 469 children, which included children reported in this study, showed a higher rate of morbidity and mortality in children compared with HICs, with young age and comorbidities being the significant predictors of severe disease.^7^

The role of coinfection requires further consideration. A reduction in the detection of respiratory viruses and seasonal variation due to SARS-CoV-2 control measures was reported worldwide during the initial months of the pandemic.^9,10,24,25^ Our study took place during the first and beginning of the second COVID-19 wave when heavy lockdown restrictions were in place, which included travel restrictions, school closure, no gatherings, social distancing and wearing of masks in public places. Despite these measures, the detection of other respiratory viruses in 66% of patients is much higher than that reported in other studies.^3,12^ RSV, HRV and adenovirus were most commonly detected, but notably there was almost no influenza. This observation emphasizes that the effect of infection control/lockdown measures on the prevalence of other viral pathogens is highly setting-specific. The catchment area of our hospital mainly consists of urban slums and informal settlements, where the effect of infection prevention measures on viral transmission is likely to be minimal.

Although no children died from ARI in our study, the risk of excess mortality from SARS-CoV-2 in LMIC settings with younger populations and less healthcare resources necessitates comprehensive public health interventions. Furthermore, the potential effects of early SARS-CoV-2 infection on the long-term lung health of children requires further study. There is growing evidence mainly from HICs of an association between viral infections such as RSV and HRV occurring in infancy and subsequent recurrent LRTIs or chronic respiratory sequelae, ranging from asthma to restrictive or obstructive lung disease.^26–29^ A significant proportion of children in our study were readmitted within 3 months for a respiratory reason. At this stage, the potential long-term impact of mild or severe SARS-CoV-2 infection is unknown, but its apparent predilection to cause severe infection in young infants suggests that follow up of these children is an area for further research to understand the long-term impact.

Although we were able to compare COVID-19 and non-COVID-19 respiratory disease and viral coinfections in a cohort of South African children, our study has several limitations. First, it is a convenience sample from a single center of admitted children, reflecting the more severely ill children. As we tried to capture as many patients as possible during the study period, we did not attempt to match SARS-CoV-2 cases with controls. Therefore, there is a discrepancy in the group sizes, which possibly influenced the analysis. Second, due to the young median age of our group, symptoms such as headache, altered taste or smell and some abdominal symptoms may have been under-reported. Lastly, we describe only the initial and beginning of the second wave of the pandemic in South Africa, and it is now known that subsequent variants of the SARS-CoV-2 virus may affect children differently.

## CONCLUSIONS

In this study, we found that among children admitted with ARI, younger age, female gender and low WAZ were associated with SARS-CoV-2 infection. SARS-CoV-2 infection was associated with significantly longer oxygen requirement. This study provides further evidence of severe COVID-19 infection among infants, with high morbidity and health resource implications despite low mortality rates. The potential impact on the long-term lung health of these babies requires further investigation.
